# Premature Baldness

**Published:** 1899-03

**Authors:** 


### Premature Baldness.

Some one said, not long ago, that the ideal symbol of faith was not the traditional maiden clinging to the Rock of Ages, but the bald-headed man confidently consulting the bald-headed specialist and faithfully looking for relief for his bald-headedness. It is a very suggestive symbol of human limitations, but when hair follicles are gone it would take a special creative act to replace them and the hirsute appendage they furnish. The treatment of premature baldness, however, is not so hopeless if it is taken in time, and skin specialists are agreed that much can be done for the condition if properly treated by prophylaxis, and early attention. In these preliminary stages, and before the real beginning of the alopecia, properly so called, the cases come into the hands of the general practitioner. Too often he is prone to make little of them, or to consider that they are inevitably progressive anyhow, and so a deformity is allowed to supervene that is unsightly, and a cause of a great deal of annoyance to the patients.

Prophylaxis is especially important. Dr. Jackson, in his Manual of Skin Diseases,* insists on two things: the influence of heredity in these cases, and the ætiological importance of dandruff. Fathers and sons for generations may grow bald early, or the inherited peculiarity may have to be traced to the grand-parents or some collateral line. Not all the children in one family in which baldness is hereditary are bald, but it will manifest itself in two or three of

*The Ready-Reference Hand-Book of Skin Diseases, by Geo. Thomas Jackson, M. D. Third Edition, just issued. Lea Brothers & Co.

the children. The necessity for prophylaxis in these cases is evident. *Hygiene of the Scalp* must begin at the very beginning of life and be continued persistently. Its details, as given by Dr. Jackson, are irksome, but most mothers whose sons are threatened with their father's early baldness, will be perfectly willing to take the additional trouble, and as for the sons themselves, as soon as they come to the years of *in*discretion (or vanity), which is generally considered to be about the age of fifteen, they can usually be depended on to take for themselves all necessary precautions to stave off the unwelcome parental inheritance.

As to dandruff, it constitutes, according to Dr. Jackson, the cause of 70 per cent. of the premature baldness that occurs. Not that every one that has dandruff will become bald; experience is against that; but it is very often true that an error in the nutrition of the sebaceous glands causes sympathetic trophic disturbances in the hair follicles, and hair production ceases. In this class of cases early treatment is of the utmost importance. Lassar's method requires the taking of a good deal of trouble on the part of the patient, but it is deservedly popular because of its frequent success. In general, however, the cure of the condition causing the dandruff, which is now considered to be, in all cases, a form of eczema, seborrhoicum, will stop the loss of hair. Persistence of treatment for months is necessary, but will nearly always be crowned with success if the condition was not too far advanced when treatment was begun. When there is absolute baldness, it is extremely doubtful if anything can make the hair grow.

